# Identifying Methods for Monitoring Foodborne Illness: Review of Existing Public Health Surveillance Techniques

**DOI:** 10.2196/publichealth.8218

**Published:** 2018-06-06

**Authors:** Rachel A Oldroyd, Michelle A Morris, Mark Birkin

**Affiliations:** ^1^ Leeds Institute for Data Analytics University of Leeds Leeds United Kingdom; ^2^ School of Geography University of Leeds Leeds United Kingdom; ^3^ School of Medicine University of Leeds Leeds United Kingdom

**Keywords:** disease, review, social media, foodborne diseases, public health, infodemiology, infoveillance, digital disease detection

## Abstract

**Background:**

Traditional methods of monitoring foodborne illness are associated with problems of untimeliness and underreporting. In recent years, alternative data sources such as social media data have been used to monitor the incidence of disease in the population (infodemiology and infoveillance). These data sources prove timelier than traditional general practitioner data, they can help to fill the gaps in the reporting process, and they often include additional metadata that is useful for supplementary research.

**Objective:**

The aim of the study was to identify and formally analyze research papers using consumer-generated data, such as social media data or restaurant reviews, to quantify a disease or public health ailment. Studies of this nature are scarce within the food safety domain, therefore identification and understanding of transferrable methods in other health-related fields are of particular interest.

**Methods:**

Structured scoping methods were used to identify and analyze primary research papers using consumer-generated data for disease or public health surveillance. The title, abstract, and keyword fields of 5 databases were searched using predetermined search terms. A total of 5239 papers matched the search criteria, of which 145 were taken to full-text review—62 papers were deemed relevant and were subjected to data characterization and thematic analysis.

**Results:**

The majority of studies (40/62, 65%) focused on the surveillance of influenza-like illness. Only 10 studies (16%) used consumer-generated data to monitor outbreaks of foodborne illness. Twitter data (58/62, 94%) and Yelp reviews (3/62, 5%) were the most commonly used data sources. Studies reporting high correlations against baseline statistics used advanced statistical and computational approaches to calculate the incidence of disease. These include classification and regression approaches, clustering approaches, and lexicon-based approaches. Although they are computationally intensive due to the requirement of training data, studies using classification approaches reported the best performance.

**Conclusions:**

By analyzing studies in digital epidemiology, computer science, and public health, this paper has identified and analyzed methods of disease monitoring that can be transferred to foodborne disease surveillance. These methods fall into 4 main categories: basic approach, classification and regression, clustering approaches, and lexicon-based approaches. Although studies using a basic approach to calculate disease incidence generally report good performance against baseline measures, they are sensitive to chatter generated by media reports. More computationally advanced approaches are required to filter spurious messages and protect predictive systems against false alarms. Research using consumer-generated data for monitoring influenza-like illness is expansive; however, research regarding the use of restaurant reviews and social media data in the context of food safety is limited. Considering the advantages reported in this review, methods using consumer-generated data for foodborne disease surveillance warrant further investment.

## Introduction

### Background

The Food Standards Agency (FSA) estimates that there are more than 1.7 million cases of foodborne illness contracted each year in the United Kingdom, of which 22,000 cases result in hospital admission and 700 cases result in death [1]. Defined as an infectious intestinal disease (IID), foodborne illness is caused by harmful pathogens such as parasites, viruses, and bacteria which enter the body through the ingestion of food or drink [2]. Symptoms include vomiting, diarrhea, fever, and abdominal pain and can vary from mildly uncomfortable to severe [3]. Although many victims recover in a few days, cases in vulnerable populations can incur lasting effects or result in fatality, especially if symptoms are persistent. Elderly people older than 65 years, young children under 5 years, pregnant women, and individuals with immunocompromising diseases are particularly at risk and are more likely to suffer from severe secondary effects such as dehydration. The burden of foodborne illness on the population and economy remains unacceptably high, and its reduction is a key objective in the FSA’s Foodborne Disease Strategy [4].

A foodborne pathogen can infect a food vehicle at any point in the supply chain, from farm to fork; however, it can be difficult to verify foodborne illness and track an infected food vehicle unless an afflicted individual visits their general practitioner (GP) and submits a sample for laboratory testing. As GP data processing takes approximately 2 weeks, an outbreak may be escalated by delay in the identification and isolation of the responsible pathogen. GP data are not only untimely but also severely underestimate the true incidence of foodborne illness as many people choose to recover at home without visiting a medical practitioner. Combined with the infrequency of sample submissions for laboratory testing, underreporting occurs at both the patient and GP level [5]. In recent years, many studies have explored the use of online consumer generated data (CGD) to undertake public health monitoring and disease surveillance. These data, which include Twitter, restaurant reviews, and Web browser searches, are thought to have many advantages over traditional data [6]. They are timelier, may have the potential to fill gaps in the reporting process, and include additional metadata appropriate for further analysis.

### Consumer-Generated Data for Disease Surveillance

Studies using CGD have ranged from influenza monitoring [5,7,8] to the surveillance of dental pain [9], but surprisingly, studies focusing on the incidence of foodborne illness are limited [10]. With the potential to improve surveillance in the food safety domain, it is important to understand and evaluate key methodologies used in CGD analysis. This review aims to identify and formally analyze primary research papers concerned with the use of CGD for disease and public health surveillance with a view to summarizing transferable methods for monitoring the outbreak and incidence of foodborne disease. It is hoped that the application of these methods may improve future policy and practice in the domain.

## Methods

### Review Question and Scope

Structured scoping methods were used to identify peer-reviewed papers, conference papers, and proceedings published between 2002 and 2017. Papers outlining methods concerned with, or transferable to, using CGD for the surveillance and monitoring of foodborne illness were of particular interest. CGD is defined as data created and made publically available by the general population. Public health is defined as the health of the population as a whole. Disease surveillance is defined as the monitoring of an illness or sickness presenting a set of well-defined symptoms.

### Search Strategy

**Figure 1:**
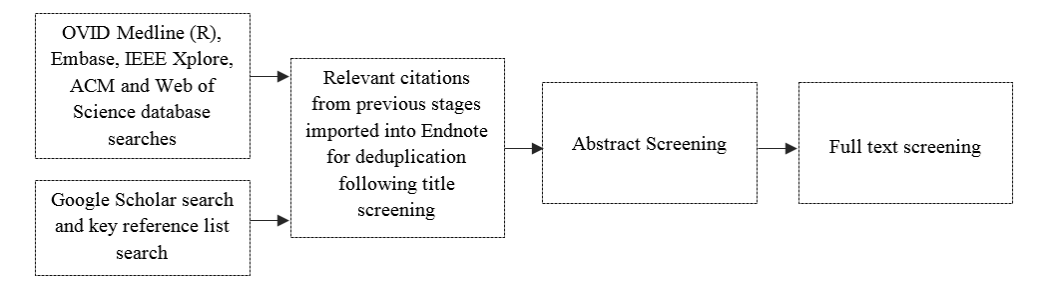
Outline of search strategy.

The abstract, title, and keyword fields of 5 individual databases were searched using predetermined search criteria. Due to the multidisciplinary nature of the review topic, the databases were specifically chosen to ensure they covered a range of discipline areas with a view to capture all relevant literature relating to disease and public health surveillance. The databases were selected to cover 3 broad topic areas: multidisciplinary (*Web of Science*); medical science (*Ovid MEDLINE, Embase*); and computing science (*IEEE Xplore, ACM*). The predetermined search terms are outlined in Table 1 and relate to 3 themes: data (eg, Twitter); application (eg, food), and methods (eg, monitor); these were adapted for each database to ensure appropriate syntax. The searches were limited to papers published after 2002 to coincide with the Web 2.0 movement. Web 2.0 describes the emergence of online communities, including the proliferation of social media, and the transition toward dynamic and user-centric Web design in the early 2000s. The search terms and the limitations detailed here were based on the methods adopted in the 2013 systematic review by Bernardo et al [10]. The full search strategy can be seen in Figure 1.

Alongside the database searches, a supplementary Google Scholar search was conducted in an attempt to capture missing literature. The search terms were *social media and infectious intestinal disease*, *restaurant review data and infectious intestinal disease*, *social media data for foodborne illness, novel data for foodborne illness, online data for food safety, social media and public health, social media and disease surveillance, online data and public health,* and *online data and disease surveillance*; these searches were limited to the top 100 most relevant hits. The titles of papers returned by Google Scholar and the database searches were screened for relevancy by one independent reviewer. Reference list reviews of key papers were also undertaken to ensure relevant publications were not missed. Subsequently, all relevant citations were imported into EndNote (Clarivate Analytics, Philadelphia), a reference management tool.

**Table 1:** Database search terms. adj4, where 2 words appear within a distance of 4 words; adj2, where the 2 words appear within a distance of 2 words. Word stems are used to ensure inflectional and derivational forms are included*.*

Search component	Search terms
Data	((micro-blog* or social media or twitter or yelp or trip advisor) adj4 ((public adj1 health) or influenza or (disease* adj1 surveillance)))
Application	((online or track or monitor) adj4 ((food*)or(illness*) or (gastroenteritis) or (influenza) or (infectious adj1 intestinal)))
Methods	(disease* or epidemic* or online or syndromic) adj2 (early or detect* or monitor* or model* or surveillance or control) Infoveillance
	natural adj2 (language or processing)
	Infodemiology

**Abstract screening exclusion criteria.**

1. Studies using non-Western data, eg, Weibo or Sino microblogs
2. Studies not written in English language
3. Studies referring to disease in nonhuman populations
4. Studies concerned with the microbiological detection of disease
5. Studies concerned with public health monitoring or disease surveillance using traditional data
6. The use of social media as a tool for patient support
7. Studies conducting sentiment analysis of social media messages
8. The use of social media as a communication tool by health care organizations
9. The use of social media by researchers to disseminate medical research findings
10. Studies profiling social media users
11. Studies examining the use of mobile phone apps for infoveillance
12. Surveillance and monitoring of mental health problems and outcomes including alcoholism and suicide
13. Surveillance of drug abuse
14. Studies of smoking cessation
15. Studies concerned with noncommunicable diseases including neurological diseases, cancer, epilepsy, psychogenic seizures, migraine, and multiple sclerosis
16. Studies using search query data such as Google Flu Trends

Following deduplication, each citation deemed relevant in the previous screening stage was subject to full-text review to determine its relevance based on predetermined exclusion criteria, outlined in Textbox 1. Papers that matched the exclusion criteria were discounted at this point. Studies using CGD, including social media data and restaurant reviews for calculating the incidence of public health or disease within the population, were considered relevant. This included published journal papers, conference papers, and proceedings. Any paper not written in English language was discounted because of the absence of resources for translation.

### Thematic Analysis

After full-text review, a thematic analysis was undertaken on those studies which were deemed relevant in an attempt to identify important methodological considerations. Data extraction was undertaken using a set of predefined criteria to ensure this process was standardized across each relevant study. Information relating to *topic, geographic region, primary data type, corpus size, control data type (if used), keyword selection, methods, results, demographic analysis, and limitations* was extracted and summarized. See Multimedia Appendix 1 for the full data characterization form.

## Results

### Data Extraction

**Figure 2:**
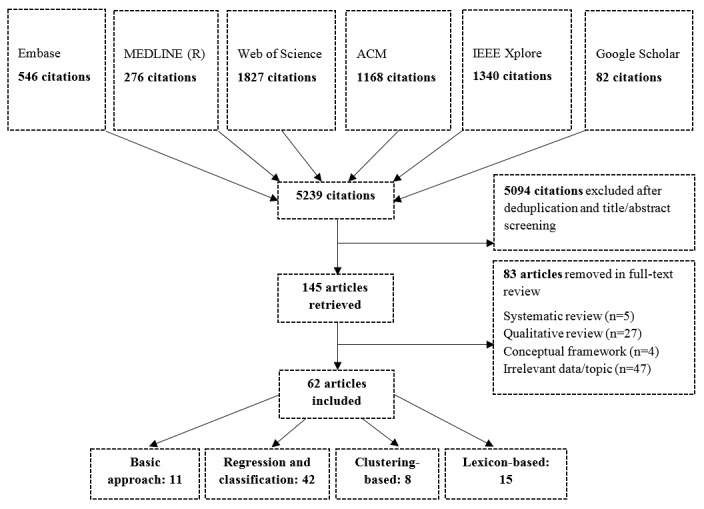
Search results. Many studies employed multiple methodological approaches.

A total of 5239 papers matched the predetermined search terms during the 5 database searches. Moreover, 82 research studies were identified during the Google Scholar search and key paper reference list search. After deduplication and title and abstract screening, 145 papers were thought to discuss the use of CGD for public health and disease monitoring, and after full-text review, 62 papers were deemed relevant to this review. See Figure 2 for an overview of this process.

### Data Characterization

Of 145 papers, 5 papers were systematic or scoping reviews of existent literature and 27 were qualitative overview or commentary papers discussing the strengths, challenges, and advances in novel data. In addition, 4 papers described conceptual and theoretical frameworks for the use of CGD in disease surveillance and 47 were deemed irrelevant on further inspection because of the topic, data, or methods used. A total of 62 papers proposed a process of primary CGD analysis to determine individual cases of public health or disease reporting and were therefore considered relevant. The full list of relevant papers is available in Multimedia Appendix 2.

The majority of relevant studies (40/62, 65%) described the use of CGD for monitoring outbreaks of influenza-like illness (ILI), 8 focused on the general topic of public health monitoring and looked at a spectrum of ailments such as allergies and back pain. Moreover, 7 studies discussed general disease including conjunctivitis and pertussis. Only 10 studies discussed the use of novel data in the domain of foodborne illness, gastroenteritis, or IID. Twitter data were the most common primary data source and were used in 58 of 62 studies. These studies used corpora between 1000 and 1 billion tweets. Of those studies which did not use Twitter data, 3 used Yelp restaurant reviews to explore food safety [11-13] and 1 study used internet forums [14].

The majority of studies in this review attempted to quantify disease or public health ailment incidence over a specific time interval by calculating the number of individuals reporting symptoms through via social media or through a restaurant review.

Moreover, 11 of 62 studies used a basic methodological approach to calculate disease incidence, whereby the occurrence of messages containing a specific keyword or number of keywords were used to represent reports of illness. In addition, 42 studies used regression or classification techniques in an attempt to filter irrelevant messages from the data corpus, and 8 studies used unsupervised clustering-based methods to identify relevant messages. Furthermore, 15 studies used lexicon-based methods to generate statistics based on term weights and term frequencies to filter relevant messages from a large data corpus.

### Thematic Analysis

A total of 4 thematic areas were identified in this review: (1) methods for calculating disease incidence using a large text corpus; (2) the challenges of working with unstructured text data; (3) the challenges of using CGD for disease surveillance; and (4) the advantages of using CGD for disease surveillance. We will discuss each theme in turn in the Discussion section of this paper.

## Discussion

### Methods for Calculating Disease Incidence

The methods used to calculate disease incidence are varied and wide-ranging in sophistication and complexity; therefore, with a view to discussing this theme with clarity, the methodological approaches have been divided into 4 broad classes: B) basic approach; R) regression and classification approach; C) clustering approach; and L) lexicon-based approach. This method categorization is based on a similar classification proposed by Witten and Frank [15], with the inclusion of an additional class for lexicon-based methods that do not fit into any of the previous classes. It should be noted that the process of categorizing text classification methods is not a menial task. Many methodological approaches can be extended to use different traits and are not discrete in their characteristics; they can, therefore, be considered as a member of more than one class.

The first class, basic approach, describes the least sophisticated method of disease incidence calculation. In some studies [11,16-27], simple keyword occurrence is used to calculate the incidence of disease in the population. As an example, Quincey and Kostkova [24] used a single keyword “flu” to collect messages from the Twitter application programming interface (API). Each tweet was assumed to represent a report of first-person illness, and an ILI rate was calculated based on the number of reports. Unlike other studies, this paper did not compare its results with a baseline measure of influenza, such as the rate reported through the Center for Disease Control (CDC) ILI Network; therefore, its performance cannot be assessed. However, studies using this crude approach generally report results that are highly correlated with published statistics (*r*>.6). In a study by Culotta [28], ILI incidence was quantified by collecting tweets that matched a small set of keywords. When compared with CDC data, the calculated rate achieved a correlation coefficient of *r*=.964, which suggests that lightweight approaches for disease surveillance cannot be dismissed. The main problem with the basic approach arises when the data are used for predictive purposes.

Krieck et al [29] state that online messages that include a specific disease name are more likely to be health-related communications or media papers than a report of illness. Therefore, models that calculate incidence based on a single disease keyword without adopting more sophisticated filtering techniques are extremely sensitive to false alarms. For example, the recall of a flu vaccine or a new government policy would lead a predictive model to detect a nonexistent rise in flu rates due to increased media coverage. Culotta [28] proceeded to analyze the robustness of lightweight methods against such false alarms by calculating the correlation of spurious keywords such as “vaccine” and “shot” with CDC ILINet data. The spurious keywords achieved similar correlations as nonspurious keywords, proving the need to use methods to filter false positive messages from the data corpus. Culotta [28] illustrates this by training a bag-of-words classifier to predict whether a message was reporting an ILI symptom or not. Although this did not significantly improve the model correlations, the application of this classifier reduced the mean-squared error from 0.077 to 0.023, reducing the model’s sensitivity to false alarms. This type of classifier falls within the second class of methodological approaches, R, as defined during data characterization. This class includes regression and classification techniques used to remove irrelevant messages and background noise.

Considered more sophisticated than the basic approach, these methods include probabilistic and generalized linear models and machine learning algorithms such as Support Vector Machine (SVM), Naïve Bayes (NB), and Decision Trees. These methods aim to reduce the size of the data corpus and calculate disease incidence only from messages that fit into the relevant class [30]. They are, therefore, more robust against false alarms compared with keyword-only approaches. The most commonly used method in this class, used in 22 studies [8,13,14,28,31-48], was SVM. SVM is a supervised, nonprobabilistic binary linear classifier. Provided with a labeled training dataset, SVM will learn a classification algorithm and assign unseen examples to a given class. Kang et al [13] used an SVM to label Yelp data in an attempt to predict hygiene violations for restaurants in Seattle. This study found that textual content such as unigram and bigram features, constructed of one- and two-word terms, respectively, are able to predict health violations with high accuracy (83%). Textual features outperformed measures such as review rating and inspection history, which achieved accuracies of 53% and 72%, respectively. Kang et al [13] found that terms such as *student*, *door*, and *the size* frequently occurred in restaurants with low hygiene scores, whereas terms referring to *selection*, *atmosphere,* and *ambiance* were indicative of a hygienic restaurant. This study suggests that factors contributing to food safety concerns can be extracted from restaurant review site messages, and highlights the capacity of text reviews as a useful indicator of food hygiene practices.

In a similar study, Kate et al [14] used SVM alongside a multinomial NB classifier to monitor food safety violation reports from internet forums. NB is the second most commonly used classifier in this methodological class, used in 13 studies [5,8,14,31,38,43,47-53]. It is a supervised classification algorithm that is probabilistic in its approach. NB, therefore, assigns new examples to a given class based on a calculated degree of certainty. When applied to the problem of filtering relevant and irrelevant messages relating to food safety violations, Kate et al [14] found that the NB model was outperformed by the SVM classifier, which achieved precision and recall values of 0.795 and 0.75, respectively. Precision and recall values for the NB model were not presented in this study; however, in comparative studies undertaken by Achrekar [5] and Carlos and Matos [31], SVM was found to achieve higher precision and recall values than NB. Although these studies suggest that SVM may be a superior classification compared with NB, it should be noted that both models are sensitive to parameter optimization. Different parameters can drastically change the results of a model; consequently, NB may perform better in a different scenario, given a different dataset and different parameters. A possible explanation for NB’s inferior performance in these particular studies is the way in which it considers terms located in the same message. When making classification decisions, SVM takes into consideration the correlations between single-term values that constitute a message, whereas NB assumes that each term contributes independently to the probability that the message is relevant or not, without considering interterm correlations [54]. Subsequently, it may be that terms within messages relating to public health and disease are more interrelated than terms in messages relating to other domains. In this case, SVM would be a more suitable technique than NB. Despite NB’s underperformance in these particular studies, one of its advantages is that it requires only a small number of training data examples to estimate the required parameters. The requirement of quality training data is considered the main limitation of all supervised text-based classification methods.

Studies using methods that fall within the second class, regression and classification, report the highest correlations with baseline measures; however, before classification can begin, they require training. Achrekar [5] used a rule-based classifier and achieved a correlation of *r=*.98, and similarly, Doan [55] used a hybrid classifier using both semantic and textual inputs and achieved a correlation of *r*=.98, but the performance of these supervised algorithms is greatly dependent on the training data. Creation of a training dataset is not a simple task and can be extremely resource-intensive [56]. Ideally, a training dataset should be representative of the real-world problem, of sufficient size to capture input-to-input and input-to-output feature relationships, and should be composed of independent examples [57]. Most studies identified in this review generated training data by manually labeling a small sample of messages [32,49], but some studies [5,33-35,50] leveraged virtual human intelligence via Amazon Mechanical Turk (AMT) for the task. AMT provides a scalable and on-demand workforce, allowing large training datasets to be generated in a less resource-intensive way. Using AMT, Achrekar [5] labeled 25,000 tweets and trained an SVM to classify relevant and irrelevant tweets relating to ILI. The provision of such a large training dataset could explain the high correlations (*r=*.98) reported by this study. Not only was AMT used to create large training datasets but it was also explored by some studies as a utility to overcome class imbalance.

Document classifiers work best when the number of messages deemed relevant and irrelevant is approximately equal. When this is not the case, eg, when only 5% of messages report foodborne illness, the classifier is biased toward the majority class in an attempt to minimize error scores. This problem is known as class imbalance. In an attempt to address class imbalance, Sadilek et al [35] used a method of human-guided machine learning, whereby instances belonging to the minority class were actively provided to the model during the training process. This study used AMT to find reports of foodborne illness, rewarding the workforce for each unique instance they found. This model achieved a precision value of 0.63 compared with data provided by the Department of Health and Mental Hygiene; however, the training dataset constituted only 200 examples. It is thought a higher precision value may have been achieved from a larger training dataset that was more representative of the testing data. Schomberg et al [58] used an alternative method to combat class imbalance. This study found that Chinese restaurants were more likely to have health code violations compared with other restaurants (25% vs 7%). Yelp reviews from Chinese restaurants were, therefore, used to train a predictive model as they were thought to contain more instances of the positive, minority class. The authors hoped to reduce the number of false positives by training the model in favorable conditions and were able to detect health violations in 78% of restaurants in the pilot study. Alongside the problems of class imbalance, many studies also discussed the challenges and importance of using discrete datasets for the training and testing process [31,37]. If overlapping data are used for both the training and testing process, the model will label data with which it was trained, overestimating its performance. Therefore, a data corpus should be large enough to split data into distinct training and testing phases.

The third methodological approach outlined in this review was class C clustering. This class outlines models that aim to identify hidden groupings and patterns within a data corpus. Clustering algorithms maximize the similarity of messages within a specific class while ensuring messages are as distinct as possible from those assigned to other classes. Many clustering models are semisupervised or unsupervised and are therefore less resource-intensive than supervised classification models, and their performance is not dependent on the provision of quality training data. Methodological approaches in this class include k-Nearest Neighbor (k-NN), Markov-Chain State modeling, and Latent Dirichlet Allocation. A total of 8 studies in this review adopted clustering techniques to filter hidden states from the text corpus [8,14,20,31,38,59-61]. Of these methods, the k-NN algorithm is considered one of the simplest machine learning algorithms as the function is approximated locally, based on the terms closest neighbors, and all computation is deferred until classification. Nargund and Natarajan [38] used a minimally supervised k-NN alongside SVM and NB to identify messages reporting first-person allergies and messages discussing allergies. The algorithm was able to determine different types of allergy, including milk, peanut, and dog allergy, and outperformed NB and SVM with reported precision and recall values of 0.864 and 0.852, respectively. Conversely, in a study to determine ILI incidence using Portuguese tweets, Carlos and Matos [31] report that SVM achieved higher precision and recall values than k-NN. An explanation of this discrepancy may lie within the nature of the classification problems. Classifying ILI tweets can be considered dichotomous as the output will belong to either the relevant or irrelevant class. As this is a binary classification task, it makes sense that SVM performs well. Alternatively, the k-NN algorithm performs best when identifying and assigning tweets to multiple hidden classes and is, therefore, more appropriate for the allergy problem outlined in the study by Nargund and Natarajan [38].

Finally, the fourth methodological approach identified in this review relates to lexicon-based approaches, class L. This class describes methods including word embeddings, term statistics, and frequent pattern mining, whereby statistics are generated based on the frequency or relative importance of a term in relation to a topic. By considering the terms that constitute a message, these models rank messages based on their overall significance. A total of 15 studies used lexicon-based methods to calculate disease incidence [14,33,34,36-38,43,44, 52,58,62-66]. For example, Velardi [62] proposed a model for the early detection of epidemics. This study weighted clusters of naïve and medical terms and assigned them to distinct classes based on their topics, for example, *cold* or *allergy*. When used to calculate rates of ILI, this model was able to achieve an extremely high correlation coefficient of *r*=.998 compared with CDC ILINet data. In another lexicon-based study, Zou et al [63] used a deep learning approach to investigate rates of IID via social media. Topical vocabulary was identified by calculating a similarity score between all word embeddings in the data corpus and the word embeddings of terms that describe IID symptoms such as *vomit* and *diarrhea*. A word embedding is defined as the words surrounding a context term. Word embeddings were ranked according to their similarity score, and those with the highest score were used to define the IID vocabulary. Zou et al [63] applied the keywords in a linear Elastic Net regression and a nonlinear Gaussian Process covariance function in an attempt to model nonlinearities between the keyword frequencies and the baseline measure of IID, as reported by Public Health England (PHE) over set time intervals. The Gaussian Process covariance function outperformed the Elastic Net regression reporting correlations of up to *r*=.77. Compared with the study undertaken by Velardi [62], this model reports relatively low correlation coefficients. However, this may be because the gold standard measure against which the IID correlations are calculated is not as representative as the gold standard measure for ILI in Velardi [62]. IID is notoriously hard to diagnose, and PHE data are based solely on laboratory-confirmed cases of pathogens. As IID is severely underreported at the patient and GP level, PHE data are not representative of the true incidence of disease. In comparison, the CDC collects data from more than 400 public health and clinical laboratories to calculate ILI rates and therefore may be considered more accurate as a baseline measure. Lexicon-based approaches are highly dependent on both the size of the vocabularies used and the similarity score threshold value beyond which the word embeddings are no longer deemed relevant to the defined topic; therefore, an alternative vocabulary and threshold value may yield differing results.

### Working With Unstructured Text Data

Although some studies used datasets from previous studies, eg, Doan et al [55] used the dataset collected by Culotta [67], many collected primary data and therefore faced the methodological challenge of preprocessing online messages into a useable format. Many studies used natural language processing (NLP) methods to remove HTML characters, emoticons, stop words, and punctuation in an attempt to filter noise from the useful part of the message. Stemming and lemmatization techniques were also used to reduce inflectional and derivational forms of a word to a common word base. Popular libraries for NLP included the Natural Language Toolkit [31,51], TextBlob [68] in Python, and the Apache OpenNLP library [64]. Alongside removing useless noise from the message, these preprocessing techniques also ensure that the data corpus is in a useful form for subsequent phases of analysis, such as the application of a document classifier.

In an attempt to filter spurious messages such as health communications and media-related tweets before disease incidence calculation, many studies removed retweets, replies, and tweets with a URL. As mentioned previously, these messages are unlikely to represent first-person accounts of disease and can increase the model’s sensitivity to false alarms. To illustrate this, Aslam et al [32] observed the correlation coefficients of 4 Twitter datasets against ILI rates published by the United States CDC—nonretweets, retweets, tweets with a URL, and tweets without a URL. Although the results differed spatially, a general trend observed was that nonretweets and tweets without a URL provided higher and more statistically significant correlations (*r>*.5) in comparison with the other 2 categories, reaffirming that retweets and tweets with a URL are not likely to represent a report of illness. Alongside removing retweets and tweets with a URL, many studies also discussed the importance of using feature selection either on data collection or as an initial filtering technique before more complex methods were undertaken. Feature selection reduces the size of the data corpus and attempts to remove messages that are highly likely to be irrelevant.

For feature selection, many studies selected only tweets that matched a keyword list of relevant terms, built in various ways. Some consulted experts in the field to generate a list of terms relating to disease symptoms [39,60], whereas others mined blogs and websites to collect terms [55,69,70]. To identify symptom-related words, Velardi et al [62] collected naïve and technical keyword pairs describing infectious disease from Google and Wikipedia. Similarly, Doan et al [55] collected syndrome terms from the BioCaster Ontology database [71], a knowledge model of layman terms. Other studies used document seeding to generate relevant keyword lists with which to select features. This process involves collecting a seed set of online messages matching 1 or 2 keywords and further expanding the keyword list to include the seed set’s most frequently occurring words. Chen et al [59] used 230 keywords defined by Chakraborty [72] and in-house experts to retrieve the seed set; the keyword set was then expanded to 2739 frequently occurring words for subsequent phases of data collection. Similarly, Culotta [7] retrieved a candidate set of tweets matching 4 keywords: *cough*, *sore throat*, *headache,* and *flu* and then selected the top 5000 frequently occurring terms. This study compared the performance of both residual sum of squares (RSS) and Pearson correlation coefficient for candidate seeding, and found that RSS performed the best. Other techniques include using the chi-square test to identify relevant trends for feature selection, adopted by Chew and Eysenbach [17], and selection based on geographical location [32,35,40,73].

### Challenges of Using Consumer-Generated Data for Disease Surveillance

The reduction of false positives and removal of spurious messages was the main methodological challenge reported by the majority of studies in this review. Although it was generally reported that high correlations against calculated results and published statistics could be achieved with a fairly crude model, these models are sensitive to increased media coverage and, therefore, prone to false alarms if used for predictive purposes [28]. Models that reported high performance and robustness against false alarms used more sophisticated methods of document classification, particularly those methods assigned to the regression and classification class (R). Although the performance of classification and regression models is highly dependent on the provision of quality training data, the collection of which can be resource intensive, they are able to achieve extremely high correlations against baseline measures compared with a basic approach, a clustering approach or a lexicon-based approach.

Related to the challenges associated with reducing false positives is the process of dealing with sarcastic and ironic messages. Greaves et al [74] state that “Sarcasm and irony, a feature of the British and US cultures, are almost impossible to process”; however, this does not negate the potential for studies using CGD for public health and disease surveillance. Indeed, a model that assumes term independence and does not consider interterm relationships such as NB is more likely to wrongly classify a sarcastic or ironic message. Alternatively, models that use a holistic approach and consider interterm relationships are better equipped to deal with sarcasm, and many methodological approaches have been proposed to deal with this problem. These methods include pattern-based approaches [75], hashtag tokenizers [76], and context incongruity [77].

A further methodological limitation of using CGD for disease surveillance is demographic representativeness. As certain demographic groups, such as elderly people, are less likely to use the internet, they are underrepresented in data derived from social media and review sites. Although this limitation is well discussed in the literature, only 8 of 62 relevant studies mentioned or undertook demographic analysis. Aslam et al [32] state that 31% of Twitter users are aged between 18 and 29 years. Broniatowski et al [41] and Carlos and Matos [31] also discussed how this age group are well represented compared with other users. Achrekar et al [5] carried out age-based ILI prediction on a small sample and found that the proposed model achieved high correlations with outbreaks among 5- to 49-year-olds but did not represent other age groups as well. These findings reaffirm that younger age groups are more prevalent on Twitter. Alternatively, Culotta [7] states the following:

...despite the fact that Twitter appears targeted to a young demographic, it in fact has quite a diverse set of users. The majority of Twitter’s nearly 10 million unique visitors in February 2009 were 35 years or older, and a nearly equal percentage of users are between ages 55 and 64 as are between 18 and 24.

There is no clear agreement on the subject, and further work is required to explore the demographic representativeness of social media and review datasets and understand the effect this has on the accuracies of models such as those discussed in this review.

### Advantages of Consumer-Generated Data

Using CGD to calculate disease incidence and public health ailments has certain advantages over traditional datasets. CGD often contains additional metadata and text, which is not available in traditional data. When writing a restaurant review, a consumer may comment on the cleanliness of the restaurant, the service, and the food they ate, providing valuable information relating to food safety procedures and the restaurant environment which can be used to inform food safety research [13]. Schomberg et al [58] used ~70,000 Yelp reviews from San Francisco to predict restaurants’ likelihood of health violation. This study labeled each review as 1 of the 3 categories depending on keyword matches: physical environment match; sentiment match; and foodborne illness match. Using the additional information in this way provided insight into other aspects of the restaurant experience, which may be helpful to health inspectors. In a similar study, Nsoesie et al [11] extracted specific food vehicles from online restaurant reviews reporting foodborne illness and ranked them in order of frequency. This study found a high correlation (.78) between the frequency of food vehicles reported in restaurant reviews and frequently occurring food vehicles in the CDC’s Foodborne Outbreak Online Database. Although not explicitly concerned with identifying cases of foodborne illness, this study outlines the importance of additional information provided via online reviews in tracking harmful pathogens in the supply chain.

Another advantage reported in almost each study was the timeliness of novel data compared with traditional data. Traditionally, public health monitoring is undertaken using GP data reported via national surveillance, which has a latency of around 2 weeks between GP appointment and data publication [78]. Due to this latency, the data are of limited use for monitoring public health outbreaks [5]. Although GP data take around 2 weeks to publish, data collected through social media or online reviews can be collected in near real time, providing a valuable resource for the timely identification and isolation of a foodborne pathogen. In addition, as many people choose to remain at home and recover from foodborne illness without visiting their GP, reports of foodborne illness identified through social media and online reviews may help to fill gaps in national surveillance data caused by underreporting at both the GP and patient level.

### Conclusions

This review identified and formally analyzed 62 primary research papers concerned with the use of CGD for public health monitoring and disease surveillance. The methodological approaches adopted by these studies were categorized into 4 broad categories: B) basic approach; R) regression and classification approaches; C) clustering approaches; and L) lexicon-based approaches and were analyzed with a view to understanding their strengths, weaknesses, and application in the domain of food safety. Only 10 research studies that used methods for monitoring foodborne illness or IID were identified. However, the methods adopted by other studies are highly transferable to the surveillance of foodborne illness, and many recommendations have emerged through the analysis of these methods.

Studies that achieved the highest and most significant correlations against published statistics adopted supervised machine learning document classifiers, the most common of which was SVM. Although the performance of document classifiers depends highly on the application and input parameters, SVM was found to be highly suitable for binary classification tasks, whereby the output is dichotomous. This includes tasks such as classifying positive and negative reports of foodborne illness. Studies using a classifier to filter false positives were found to be more robust against false alarms than studies adopting a basic approach based on keyword incidence. Feature selection was also found to improve the performance of the model by removing messages deemed unlikely to be relevant before classification. Of the feature selection techniques, filtering messages using symptom-specific keyword lists based on existing knowledge mined from blogs and websites was the most suitable. This type of keyword list was more likely to retrieve messages reporting illness compared with disease-specific keywords such as “food poisoning.”

The demographic limitations of CGD are unclear, and future work should focus on understanding the effect of these limitations on model outcomes. Demographic limitations were only discussed in a handful of reviews. However, provisional findings show that people aged between 18 and 29 years are well represented on Twitter but are underrepresented in national foodborne illness outbreak statistics, as they prefer to recover at home without seeking medical advice from their GP. This highlights the utility of CGD to complement traditional data sources. The lack of primary research in the area of CGD for food safety provides a strong case for further research. Considering the reported success of studies in other health-related fields, it is thought that CGD could prove useful in helping to inform and improve current inspection procedures in the United Kingdom by identifying problematic restaurants and specific outbreaks of disease. In the long term, a model that can successfully detect reports of foodborne illness through social media data and online restaurant reviews could reduce the burden on the economy and, more importantly, the population. CGD may also have the capacity to fill gaps in national surveillance data and combat problems associated the underestimation of disease incidence.

## Appendix

Multimedia Appendix 1 Data characterization form used to extract relevant information during full-text review.

Multimedia Appendix 2 Results of data characterization and methods coding (Alco: alcohol sales; D: disease; FBI: foodborne Illness; ILI: influenza-like illness; IID: infectious intestinal disease; PH: public health). Coding: ARX: autoregressive modeling with exogenous terms; BOLASSO: bootstrapped least absolute shrinkage and selection operator; BOW: bag of words; DT: decision tree; k-NN: K-nearest neighbor; LASSO: least absolute shrinkage and selection operator: LDA: latent dirichlet allocation; NB: naïve Bayes; PDE: partial differential equation; POS: part of speech tagging; RF: random forest; SVM: support vector machine; TS: term statistics).

## Abbreviations

AMT: Amazon Mechanical Turk
CDC: Center for Disease Control
CGD: consumer generated data
FSA: Food Standards Agency
IID: infectious intestinal disease
ILI: influenza-like illness
GP: general practitioner
k-NN: k-Nearest Neighbor
NB: Naïve Bayes Classifier
NLP: natural language processing
PHE: Public Health England
RSS: residual sum of squares
SVM: support vector machine
