# Supplementary material for: Identifying Methods for Monitoring Foodborne Illness: Review of Existing Public Health Surveillance Techniques
Source: JMIR Public Health Surveill. 2018 Jun 6;4(2):e57. doi: 10.2196/publichealth.8218 (PMC6010836; doi:10.2196/publichealth.8218)
Supplement: Multimedia Appendix 1 [file publichealth_v4i2e57_app1.pdf]

Multimedia Appendix 1. Data Characterisation form using during full text review.

Data Characterisation	Additional Information
Topic	State topic of paper:<br>ILI = Influenza Related Illness<br>FBI = Food Borne Illness<br>IID = Infectious Intestinal Disease<br>Disease<br>H1N1<br>Public Health
Country	State country of data collection
Primary data type	State data type:<br>Twitter<br>Online restaurant review<br>Other
Control data type	State if gold standard measure was used for comparison, e.g.:<br>PHE foodborne disease outbreak data<br>Centre for Disease Control ILI Network
Keyword selection	State how keywords were generated for data collection:<br>Single Keyword<br>Keyword List<br>Manually generated<br>Automatically generated<br>Knowledge based generation (mining blogs / webpages etc.)
Methods	State methods used for identifying cases of disease / ailment<br>Basic keyword matching<br>Keyword / lexicon based analysis (term frequencies etc.)<br>Semantic analysis (based on non-textual features)<br>Machine learning classifier (SVM, NB etc.)<br>Clustering / topic modelling (LDA etc.)<br>Custom classifier<br><br>State performance of methods:<br>Precision and recall.<br><br>For supervised classifiers:<br>Size of training dataset<br>Process of generation (Manual labelling / Amazon Mechanical Turk etc.)
Results	State results of incidence calculation:<br>Correlation coefficients (if calculated)
Demographic analysis	State if the paper discussed the demographic limitations of using CGD for disease / ailment surveillance.
Limitations	Limitations of the study as outlined by the author, e.g. sample size.<br>Limitations of the study as identified by reviewer.
