# Supplementary material for: Identifying Methods for Monitoring Foodborne Illness: Review of Existing Public Health Surveillance Techniques
Source: JMIR Public Health Surveill. 2018 Jun 6;4(2):e57. doi: 10.2196/publichealth.8218 (PMC6010836; doi:10.2196/publichealth.8218)
Supplement: Multimedia Appendix 2 [file publichealth_v4i2e57_app2.pdf]

Appendix 2 – Results of data characterization and methods coding

Author	*Topic*	*Data*	*Collection method*	*Approach*	*Forecasting*
[15]	ILI	4.7m tweets	Keyword list	B: keyword occurrence	Logistic ARX
[5]	ILI	4.9m tweets	Keyword list	R: rule-based classifier, DT, NB	Logistic ARX
[39]	ILI	1k tweets	Single keyword / geographic location	L: term statistics<br>R: SVM	-
[7]	ILI	400k tweets	Single keyword	R: rule-based classifier, logistic regression, SVM, NB, RF<br>C: k-NN	-
[32]	ILI	159k tweets	Single keyword / geographic location	R: probabilistic classifier, SVM	-
[59]	D	11m tweets	Keyword list	R: rule-based classifier (POS tagging)	-
[58]	ILI	300m tweets	Keyword list	R: SVM , rule-based classifier, logistic regression	Autocorrelation
[27]	ILI	14m tweets	Keyword list	R: SVM, NB, RF, DT<br>C: k-NN	Linear regression model
[41]	ILI	34k tweets	Keyword list	C: HFSTM probabilistic topic modelling (Markov / LDA)<br>Expectation Maximisation Algorithm	LASSO linear regression
[40]	ILI	34k tweets	Automatic keyword generation	C: HFSTM probabilistic topic modelling (Markov / LDA)<br>Expectation Maximisation Algorithm	LASSO Linear Regression
[16]	ILI	2m tweets	Chi-squared	B: keyword occurrence	-
[42]	ILI	121m tweets	Single keyword	C: spectral clustering, k-means clustering, PDE modelling	-
[17]	ILI	Tweets unknown	Keyword list	B: keyword occurrence	-
[60]	ILI	570m tweets	Keyword list	R: probabilistic classifier, rule-based classifier	Linear regression
[6]	ILI	574k tweets	Simple and Multiple Linear Regression	R: probabilistic classifier, rule-based classifier	BOW classifier with linear regression
[25]	ILI, Alco	570m tweets	Keyword list	R: logistic regression, SVM, DT	Linear regression<br>Support vector regression
[46]	ILI	1k tweets	Keyword list	R: rule based classifier, NB	-
[61]	ILI	2.2k tweets	Knowledge based	R: NB<br>L: word embedding	-
[18]	D	6.5k tweets	Keyword list	B: keyword occurrence	-
[49]	D	636 texts and pseudo-tweets	Expert generated keywords	R: rule-based classifier (POS tagging), SVM	-
[19]	D	450k tweets	Keyword list	B: keyword occurrence<br>C: LDA	-

[29]	ILI	587m tweets	Simple and Multiple Linear Regression / Knowledge	R: rule based classifier	-
[20]	ILI	Tweets unknown	Keyword list	B: keyword occurrence	-
[63]	ILI	Tweets unknown	Keyword list	L: Markov Chain State based on BOW	Gaussian based
[21]	FBI	2.2k tweets	Keyword list	B: keyword occurrence	-
[11]	FBI	294k Yelp reviews	Keyword list	R: probabilistic classifier	-
[65]	ILI	2.7k tweets	Keyword list	R: SVM	-
[66]	ILI	8.6 m tweets	Keyword list	R: probabilistic classifier	Linear regression with ridge regularisation
[12]	FBI	152k Yelp reviews	Restaurant reviews	R: semantic classifier , SVM	-
[13]	FBI	14.7k forum posts	Restaurant reviews	R: rule-based and semantic classifier, SVM, NB<br>C: k-NN<br>L: term statistics	-
[31]	ILI	4k tweets	Keyword list	R: rule based classifier, NB	-
[67]	ILI	160k daily tweets	Manual & automatic knowledge based	R: probabilistic weighted classifier (flu-score), LASSO	Linear regression
[68]	ILI	200k daily average tweets	Automatic knowledge based	R: probabilistic weighted classifier (flu-score), BOLASSO	-
[69]	PH	6.3m tweets	Keyword list	R: SVM, NB, RF<br>L: term statistics, POS tagging	-
[70]	D	37.5k tweets	Keyword list	R: probabilistic classifier	-
[52]	ILI	3k tweets	Geographical location	R: rule-based classifier	Autoregressive forecasting
[22]	ILI, D	170k tweets	Keyword list / geographical location	B: keyword occurrence	-
[43]	PH	430k tweets	Single keyword	R: SVM, NB<br>C: k-NN<br>L: POS tagging	-
[10]	FBI	4k Yelp reviews	Restaurant reviews	B: keyword occurrence	-
[71]	ILI	1.6m tweets	Keyword list	R: SVM<br>L: frequent pattern analysis	-
[34]	PH	2 billion tweets	Keyword list	R: SVM<br>L: LDA	-
[37]	PH	2 billion tweets	Knowledge based generation	R: SVM<br>L: LDA	-

[33]	ILI	2m tweets	Knowledge based keyword generation	R: SVM<br>L: LDA	-
[23]	ILI	135k tweets	Single keyword	B: keyword occurrence	-
[48]	D	100m tweets	Knowledge based generation	L: POS tagging, Temporal Topic Modelling	-
[35]	FBI	3.8m tweets	Geographical location	R: SVM	-
[53]	FBI	16k average daily tweets	Geographic location	R: SVM	-
[72]	ILI	Tweets unknown	Keyword list	R: Stacked linear regression, SVM, Adaboost - decision tree regression	Logistic ARX forecasting
[73]	ILI	14m tweets	Keyword list	R: NB	-
[38]	FBI	71k Yelp reviews	Keyword list / semantic features	L: term statistics	Logistic regression
[75]	ILI	950k tweets	Keyword list	R: Support Vector Regression	-
[76]	ILI	6k tweets	Keyword list	R: SVM	-
[77]	ILI	12k tweets	Keyword list	L: word embedding	-
[78]	ILI, IID	84.5m tweets	Knowledge based generation	R: SVM, NB	Autoregressive forecasting
[44]	ILI	240m tweets	Knowledge based keyword generation	L: word embedding, term statistics	-
[62]	ILI	2.9mtweets	Single Keyword	B: keyword occurrence	Partial Differential Equation analysis
[36]	PH	261m tweets	Keyword list	R: NB	-
[24]	PH	7.5m tweets	Hashtag	B: keyword occurrence	-
[47]	PH	46m tweets	Keyword list	R: probabilistic classifier, rule-based classifier	-
[64]	PH, ILI	Tweets unknown	Keyword list	R: rule-based classifier	Logistic regression
[45]	IID	585m tweets	Automatically generated keyword list	R: Elastic Net Regression, Gaussian process covariance function<br>L: word embedding	-
[74]	ILI	13.5m tweets.	Keyword list	R: SVM, multinomial logistic regression, NB	-

References

5. Achrekar H, Gandhe A, Lazarus R, Yu S, Liu B. Twitter Improves Seasonal Influenza Predictions. Proceedings of the 2012 International Conference in Health Informatics; 2012 Feb 1-4; Algarve, Portugal: 61-70.

6. Culotta A. Towards detecting influenza epidemics by analyzing Twitter messages. Proceedings of the first workshop on social media analytics; 2010 July 25-18; Washington DC, USA: 115-122. DOI: 10.1145/1964858.1964874

7. Aramaki E, Maskawa S, Morita M. Twitter catches the flu: detecting influenza epidemics using Twitter. Proceedings of the conference on empirical methods in natural language processing; 2011 July 27-31; Edinburgh, UK: 1568-1576. ISBN: 978-1-937284-11-4

10. Nsoesie EO, Kluberg SA, Brownstein JS. Online reports of foodborne illness capture foods implicated in official foodborne outbreak reports. Prev Med 2014; 67: 264-9. PMID: 25124281

11. Harrison C, Jorder M, Stern H, Stavinsky F, Reddy V, Hanson H, Waechter H, Lowe L, Gravano L, Balter S. Using online reviews by restaurant patrons to identify unreported cases of foodborne illness - New York City, 2012-2013. Morbidity and Mortality Weekly Report 2014; 63(20): 441-445. https://www.cdc.gov/MMWr/preview/mmwrhtml/mm6320a1.htm. Archived at: http://www.webcitation.org/6wvqBmzTU

12. Kang JS, Kuznetsova P, Choi Y, Luca M. Using Text Analysis to Target Government Inspections: Evidence from Restaurant Hygiene Inspections and Online Reviews. 2013. Harvard Business School: Cambridge, Massachusetts. 1-5. http://www.hbs.edu/faculty/Publication%20Files/Luca_Inspections_328fbdfc-4cbe-4b8e-8549-3a21c7a56279.pdf. Archived at: http://www.webcitation.org/6rEwdT6kB

13. Kate K, Negi S, Kalagnanam J. Monitoring food safety violation reports from internet forums. Studies in health technology and informatics 2014; 205: 1090-1094. PMID: 25160357

15. Achrekar H, Gandhe A, Lazarus R, Yu S, Liu B. Predicting flu trends using Twitter data. The First International Workshop on Cyber Networking Systems; Apr 10-15, 2011; Shanghai. 2011. DOI: 10.1109/INFCOMW.2011.5928903

16. Chew C, Eysenbach G. Pandemics in the Age of Twitter: Content Analysis of Tweets during the 2009 H1N1 Outbreak. PLoS One 2010; 5(11). e14118. PMID: 21124761

17. Chorianopoulos K, Talvis K, Flutrack.org: Open-source and linked data for epidemiology. Health Inform J 2016; 22(4): 962-974. PMID: 26351261

18. Deiner MS, Lietman TM, McLeod SD, Chodosh J, Porco TC. Surveillance Tools Emerging From Search Engines and Social Media Data for Determining Eye Disease Patterns. JAMA Ophthalmol 2016; 134(9): 1024-1030. PMID: 27416554

19. Diaz-Aviles E, Stewart A. Tracking Twitter for Epidemic Intelligence. Proceedings of the 4th Annual ACM Web Science Conference; 2012 June 22-24; Evanston, Illinois: 82-85. ISBN: 978-1-4503-1228-8

20. Gesualdo F, Stilo G, Agricola E, Gonfiantini MV, Pandolfi E, Velardi P, Tozzi AE. Influenza-Like Illness Surveillance on Twitter through Automated Learning of Naive Language. PLoS ONE 2013; 8(12): e82489. PMID: 24324799

21. Harris JK, Mansour R, Choucair B, Olson J, Nissen C, Bhatt J. Health Department Use of Social Media to Identify Foodborne Illness - Chicago, Illinois, 2013-2014. Morbidity and Mortality Weekly Report 2014; 63(32): 681-685. PMID: 25121710

22. Nagel AC, Tsou MH, Spitzberg BH, An L, Gawron JM, Gupta DK, Yang JA, Han S, Peddecord KM, Lindsay S, Sawyer MH. The Complex Relationship of Realspace Events and Messages in Cyberspace: Case Study of Influenza and Pertussis Using Tweets. J Med Internet Res 2013; 15(10): 263-275. PMID: 24158773

23. Quincey ED, Kostkova P. Early Warning and Outbreak Detection Using Social Networking Websites: The potential of Twitter. In: Kostkova P, editor. Electronic Healthcare. Springer: Berlin, Heidelberg; 2010. p. 21-24. ISBN: 978-3-642-11745-9

24. Yom-Tov E, Borsa D, Cox IJ, McKendry RA. Detecting disease outbreaks in mass gatherings using Internet data. J Med Internet Res 2014; 16(6): e154. PMID: 24943128

25. Culotta A. Lightweight methods to estimate influenza rates and alcohol sales volume from Twitter messages. Lang Resour Eval 2013; 47(1): 217-238. DOI: 10.1007/s10579-012-9185-0

27. Carlos J. Matos S, Predicting Flu Incidence from Portuguese Tweets. Proceedings of the International Work-Conference on Bioinformatics and Biomedical Engineering; 2013 March 18-20; Granada, Spain.

29. Doan S, Ohno-Machado L, Collier N. Enhancing Twitter Data Analysis with Simple Semantic Filtering: Example in Tracking Influenza-Like Illnesses, 2012. Proceedings of the 2012 IEEE Second International Conference on Healthcare Informatics, Imaging and Systems Biology; 2012 Sept 27-28; California, USA. 62-71. DOI: 10.1109/HISB.2012.21

31. Khan MAH, Iwai M, and Sezaki K. A robust and scalable framework for detecting self-reported illness from twitter. Proceedings of the IEEE 14th International Conference on e-Health Networking, Applications and Services; 2012 Oct 10-13, Beijing, China. DOI: 10.1109/HealthCom.2012.6379425

32. Aslam AA, Tsou MH, Spitzberg BH, An L, Gawron JM, Gupta DK, Peddecord KM, Nagel AC, Allen C, Yang JA, Lindsay S. The Reliability of Tweets as a Supplementary Method of Seasonal Influenza Surveillance. J Med Internet Res 2014; 16(11):e250. PMID: 25406040

33. Paul MJ, Dredze M. Discovering Health Topics in Social Media Using Topic Models. PLoS ONE, 2014; 9(8): e103408. DOI: 10.1371/journal.pone.0103408

34. Paul MJ, Dredze M. A model for mining public health topics for twitter. Proceedings of the 5th International AAAI Conference on Web blogs and Social Media; 2011 July 17-21. Barcelona, Spain. ISBN 978-1-57735-505-2

35. Sadilek A, Brennan S, Kautz H, Silenzio V. nEmesis: Which Restaurants Should You Avoid Today? Proceedings of the first AAAI Conference on Human Computation and Crowdsourcing; 2013 November 7-9; Palm Springs, California. ISBN 978-1-57735-607-3

36. Yin Z, Fabbri D, Rosenbloom ST, Malin B. A Scalable Framework to Detect Personal Health Mentions on Twitter. J Med Internet Res 2015; 17(6): e138. PMID: 26048075

37. Paul MJ, Dredze, M. You Are What You Tweet: Analyzing Twitter for Public Health. Proceedings of the 5th International AAAI Conference on Web blogs and Social Media; 2011 July 17-21. Barcelona, Spain. ISBN 978-1-57735-505-2

38. Schomberg JP, et al. Supplementing Public Health Inspection via Social Media. PLoS ONE 2016; 11(3): e0152117. DOI: 10.1371/journal.pone.0152117

39. Allen C, Tsou MH, Aslam A, Nagel A, Gawron JM. Applying GIS and Machine Learning Methods to Twitter Data for Multiscale Surveillance of Influenza. PLoS ONE 2016; 11(7): e0157734. PMID: 27455108

40. Chen LZ, Tozammel Hossain KSM, Butler P, Ramakrishnan N, Prakash BA. Syndromic surveillance of Flu on Twitter using weakly supervised temporal topic models. Data Min Knowl Discov 2016; 30(3): 681-710. DOI: 10.1007/s10618-015-0434-x

41. Chen LZ, Tozammel Hossain KSM, Butler P, Ramakrishnan N, Prakash BA. Flu Gone Viral: Syndromic Surveillance of Flu on Twitter Using Temporal Topic Models. Proceedings of the 2014 IEEE International Conference on Data Mining; 2014 December 14-17. Shenzhen, China : 755 – 760. DOI: 10.1109/ICDM.2014.137

42. Chon J, Raymond R, Wang HY, Wang F. Modeling Flu Trends with Real-Time Geo-tagged Twitter Data Streams. Proceedings of the 10th International Conference on Wireless Algorithms, Systems, and Applications; 2015 August 20-12. Qufu, China: 60-69. ISBN 978-3-319-21837-3

43. Nargund K, Natarajan S. Public Health Allergy Surveillance Using Micro-blogs. Proceedings of the 2016 International Conference on Advances in Computing, Communications and Informatics; 2016 September 21-24. Jaipur, India. DOI: 10.1109/ICACCI.2016.7732248

44. Velardi P, Stilo G, Tozzi AE, Gesualdo F. Twitter mining for fine-grained syndromic surveillance. Artif Intell Med 2014; 61(3): 153-163. PMID: 24613716

45. Zou B, Lampos V, Gorton R, Cox JI. On Infectious Intestinal Disease Surveillance using Social Media. Proceedings of the 2016 Digital Health Conference; 2016 April 11-13; Montreal, Canada. ISBN 978-1-4503-4224-7

46. Dai X, Bikdash M. Hybrid Classification for Tweets Related to Infection with Influenza, Proceedings of the IEEE Southeastcon; 2015 April 9 -12; Fort Lauderdale, Florida. 1-5. DOI: 10.1109/SECON.2015.7133015

47. Zaldumbide J, Sinnott RO, Identification and Validation of Real-Time Health Events through Social Media. Proceedings of the 2015 IEEE International Conference on Data Science and Data Intensive Systems; 2015 December 11-13; Sydney, Australia. ISBN: 9781509002153

48. Romano S, Martino SD, Kanhabua N, Mazzeo A, Nejdl W. Challenges in Detecting Epidemic Outbreaks from Social Networks. Proceedings of the 30th International Conference on Advanced Information Networking and Applications Workshops; 2016 Mar 23-25; Crans-Montana, Switzerland. Electronic ISBN: 978-1-5090-2461-2

49. Denecke K, Krieck M, Otrusina L, Smrz P, Dolog P, Nejdl W, Velasco E. How to exploit twitter for public health monitoring? Methods Inf. Med. 2013; 52(4): 326-39. PMID: 23877537

52. Nagar R, Yuan Q, Freifel, CC, Santillana M, Nojima A, Chunara R, Brownstein JS. A case study of the New York City 2012-2013 influenza season with daily geocoded Twitter data from temporal and spatiotemporal perspectives. J Med Internet Res 2014; 16(10): e236. PMID: 25331122

53. Sadilek A, Kautz H, DiPrete L, Labus B, Portman E, Teitel J, Silenzio J. Deploying nEmesis: Preventing Foodborne Illness by Data Mining Social Media. Proceedings of the fourth AAAI Conference on Human Computation and Crowdsourcing; 2016 Oct 30- Nov 3; Phoenix, Arizona, USA. ISBN 978-1-57735-774-2

58. Broniatowski DA, Paul MJ, Dredze M. National and Local Influenza Surveillance through Twitter: An Analysis of the 2012-2013 Influenza Epidemic. Plos One 2013; 8(12): e83672. DOI: 10.1371/journal.pone.0083672

59. Barros J. Text Mining from Social Media for Public Health Applications. Proceedings of the 2017 International Conference on Digital Health; 2017 July 2-5; London, United Kingdom. DOI: 10.1145/3079452.3079475

60. Culotta A. Detecting influenza outbreaks by analyzing Twitter messages. Proceedings of the First Workshop on Social Media Analytics; 2010 July 25-28; Washington DC, USA. DOI: 10.1145/1964858.1964874

61. Dai X, Bikdash M, Meyer B. From social media to public health surveillance: Word embedding based clustering method for twitter classification. Proceedings of the IEEE SouthEast Con; 2017 March 30- April 2; Charlotte, NC, USA. DOI: 10.1109/SECON.2017.7925400

62. Wang F, Wang H, Kuai X, Raymond R, Chon J, Fuller, S, Debruyn, A. Regional Level Influenza Study with Geo-Tagged Twitter Data. J Med Syst 2016; 40(8): 1-8. PMID: 27372953

63. Grover S, Aujla GS. Twitter Data Based Prediction Model for Influenza Epidemic. Proceedings of the 2nd International Conference on Computing for Sustainable Global Development; 2015 March 11-13; New Dehli, India. ISBN: 978-9-3805-4416-8

64. Zhao L, Chen F, Lu CT, Ramakrishnan N. SimNest: Social Media Nested Epidemic Simulation via Online Semi-Supervised Deep Learning. Proceedings of 2015 IEEE International Conference on Data Mining; 2015 November 14-17; Atlantic City, NJ, USA. PMID: 27453696

65. Hartley DM, Giannini CM, Wilson S, Frieder O, Margolis PA, Kotagal UR, White DL, Connelly BL, Wheeler DS, Tadesse DG, Macaluso M. Coughing, sneezing, and aching online: Twitter and the volume of influenza-like illness in a paediatric hospital. PLoS ONE 2017; 12(7): e0182008. DOI: 10.1371/journal.pone.0182008

66. Hirose H, Wang L. Prediction of Infectious Disease Spread Using Twitter: A Case of Influenza. Proceedings of the 2012 Fifth International Symposium on Parallel Architectures, Algorithms and Programming; 2012 December 17-20; Taipei, Taiwan. ISBN: 9781467345668

67. Lampos V, Cristianini N. Tracking the flu pandemic by monitoring the social web. Proceedings of the 2010 2nd International Workshop on Cognitive Information Processing; 2010 June 14-16; Elba, Italy. DOI: 10.1109/CIP.2010.5604088

68. Lampos V, and Cristianini . Nowcasting Events from the Social Web with Statistical Learning. ACM Transactions on Intelligent Systems and Technology 2012; 3(4): 2-22. DOI: 10.1145/2337542.2337557

69. Lee K, Agrawal A, Choudhary A. Mining Social Media Streams to Improve Public Health Allergy Surveillance. Proceedings of the 2015 IEEE/ACM International Conference on Advances in

Social Networks Analysis and Mining; 2015 August 25-28; Paris, France. DOI: 10.1145/2808797.2808896

70. Lim S, Tucker CS, Kumara S. An unsupervised machine learning model for discovering latent infectious diseases using social media data. J Biomed Inform 2017; 66: 82-94. PMID: 28034788

71. Parker J, Wei Y, Yates A, Frieder O, Goharian N. A framework for detecting public health trends with Twitter. Proceedings of the 2013 IEEE/ACM International Conference on Advances in Social Networks Analysis and Mining; 2013 August 25-28; Niagra, Ontario, Canada. DOI: 10.1145/2492517.2492544

72. Santillana M, Nguyen AT, Dredze M, Paul MJ, Nsoesie EO, Brownstein JS. Combining Search, Social Media, and Traditional Data Sources to Improve Influenza Surveillance. PLoS Comput Biol 2015; 11(10): 1-15. DOI: 10.1371/journal.pcbi.1004513

73. Santos J, Matos S. Analysing Twitter and web queries for flu trend prediction. Theor Biol Med Model 2014; 11(Suppl 1): S6. DOI: 10.1186/1742-4682-11-S1-S6

74. Zuccon G, Khanna S, Nguyen A, Boyle J, Hamlet M, Cameron M. Automatic detection of tweets reporting cases of influenza like illnesses in Australia. Health Inf Sci Syst 2015; 3(Suppl 1): S4. PMCID: PMC4383056

75. Signorini A, Segre AM, Polgreen PM. The Use of Twitter to Track Levels of Disease Activity and Public Concern in the U.S. during the Influenza A H1N1 Pandemic. PLoS ONE 2011; 6(5): 1-10. DOI: doi.org/10.1371/journal.pone.0019467

76. Sofean M, Smith M, Mantas J, Anderse SK, Mazzoleni MC, Blobel B, Quaglini S, Moen A. A Real-Time Disease Surveillance Architecture Using Social Networks. Stud Health Technol Inform 2012; 180: 823-827. PMID: 22874307

77. Talvis K, Chorianopoulos K, Kermanidis KL. Real-time monitoring of flu epidemics through linguistic and statistical analysis of Twitter messages. Proceedings of the 9th International Workshop on Semantic and Social Media Adaptation and Personalization; 2014 November 6-7; Corfu, Greece. PMID: 22874307

78. Thapen N, Simmie D, Hankin C, Gillard J. DEFENDER: Detecting and Forecasting Epidemics Using Novel Data-Analytics for Enhanced Response. PLoS ONE 2016; 11(5): e0155417. DOI: 10.1371/journal.pone.0155417
